# The impact of SARS-CoV-2 infection on immunity reconstitution among pediatric patients after allogeneic hematopoietic stem cell transplantation: a propensity score-matched analysis

**DOI:** 10.3389/fped.2024.1489648

**Published:** 2024-11-21

**Authors:** Xin Wang, LiPeng Liu, Luyang Zhang, Yue Shang, Xia Chen, Yuanyuan Ren, Fang Liu, Wenbin An, Yang Wan, Xiaolan Li, Wenyu Yang, Xiaofan Zhu, Ye Guo

**Affiliations:** ^1^ State Key Laboratory of Experimental Hematology, National Clinical Research Center for Blood Diseases, Haihe Laboratory of Cell Ecosystem, Institute of Hematology & Blood Diseases Hospital, Chinese Academy of Medical Sciences & Peking Union Medical College, Tianjin, China; ^2^ Tianjin Institutes of Health Science, Tianjin, China

**Keywords:** COVID-19, hematopoietic stem cell transplantation, immune reconstitution, pediatrics, virus diseases

## Abstract

**Background:**

Immunity reconstitution (IR) is crucial for pediatric patients undergoing hematopoietic stem cell transplantation (HSCT), but the impact of severe acute respiratory syndrome coronavirus 2 (SARS-CoV-2) infection on lymphocyte subsets post-transplant remains unclear. Therefore, we assessed immune cell dynamics in children after SARS-CoV-2 infection.

**Methods:**

We enrolled 42 children, including 21 post-HSCT SARS-CoV-2 infected and 21 matched, non-infected historical controls (1:1 matching based on propensity scores). The time from HSCT to SARS-CoV-2 infection in the infected group was determined by the beginning of follow-up for the non-infected group. The primary endpoint was 270-day IR kinetics post-infection.

**Results:**

Our findings showed similar recovery trends between the infected group and non-infected group both in UCB and HID recipients. In the UCB cohort, the NK cell reconstitution in the infected group was poorer compared to the non-infected group, but this difference did not reach statistical significance (*P* = 0.178). Furthermore, HID transplantation might be a trend towards poor CD19+ T-cell reconstitution [hazard ratio (HR): 0.43, 95% CI: 0.18–1.04, *p* = 0.06]. No statistically significant difference was observed in terms of secondary infections across the UCB (*P* = 0.150) and HID (*P* = 0.980) cohorts as well as there was no discernible difference in overall survival between the two groups (*P* = 1).

**Conclusions:**

Our analysis reveals that SARS-CoV-2 might temporarily impaired the IR process in the short term, with recovery to a comparable trend as observed in non-infected patients approximately 9 months post-infection.

## Introduction

Allogeneic hematopoietic stem cell transplantation (allo-HSCT) holds the potential to cure hematological malignancies and non-malignant conditions. However, this procedure is associated with numerous life-threatening risks, including transplantation-related mortality (TRM), serious infections, acute and chronic graft vs. host disease (GVHD), relapse and others (1). Delayed immune reconstitution (IR) primarily contributes to the occurrence of these complications, and the speed of this process is influenced by several factors, such as age, stem cell source, human leukocyte antigen (HLA) barriers and opportunistic infections (2). The HLA disparity in haploidentical donors (HID) and the low lymphocyte count in unrelated cord blood (UCB) graft may potentially affected IR following alternative donor transplantation (3).

Since December 2019, the severe acute respiratory syndrome coronavirus 2 (SARS-CoV-2) has spread rapidly across the world, with lymphopenia reported as its hallmark. Lymphocytes, due to their high expression of the angiotensin-converting enzyme 2 (ACE2) receptor, are susceptible to invasion by SARS-CoV-2, ultimately leading to lysis (4). According to relevant data from the Center for International Blood and Marrow Transplant Research, 42% of allo-HSCT recipients who contract Coronavirus Disease 2019 (COVID-19) suffered from moderate or severe disease, with a 30-day survival rate of 68% (5). Therefore, COVID-19 is considered particularly hazardous in the context of allo-HSCT. Furthermore, the “cytokine storm” triggered by infection, characterized by elevated levels of interleukins and tumor necrosis factor-alpha (TNF-α), likely plays a significant role in lymphocyte apoptosis (6). Lymphopenia may contribute to long COVID syndrome and be a factor in secondary infections and mortality among post-HSCT pediatric patients (7–9). However, the impact of COVID-19 on IR and subsequent outcomes in pediatric patients receiving transplants from different donors remains unclear.

In this study, we evaluated the effects of COVID-19 on IR, specifically focusing on the dynamics of immune cell numbers. Additionally, we will investigate the cumulative incidence of secondary infections and overall survival (OS) following SARS-CoV-2 infection.

## Materials and methods

### Patients and study design

A retrospective longitudinal study was designed to investigate the influence of SARS-CoV-2 infection on dynamic changes in the immune cells of children who did not achieve IR post-HSCT. We selected patients who met the following criteria: (1) were aged under 18, (2) had COVID-19 within one year after undergoing transplantation, and (3) had received either HID or UCB transplantation. From December 10, 2022 to January 17, 2023, a total of 21 pediatric patients were enrolled. The diagnostic criteria and clinical classification of COVID-19 have been described in other publications (10, 11). Throat swab samples of all hospitalized patients were tested 2019-nCoV nucleic acid using RT-PCR once or twice a week to accurately determine the SARS-CoV-2 infection. Although the omicron variant was widely prevalent in China during the study period, the specific strains infecting the pediatric patients included in this study were not explicitly identified.

**Figure 1:**
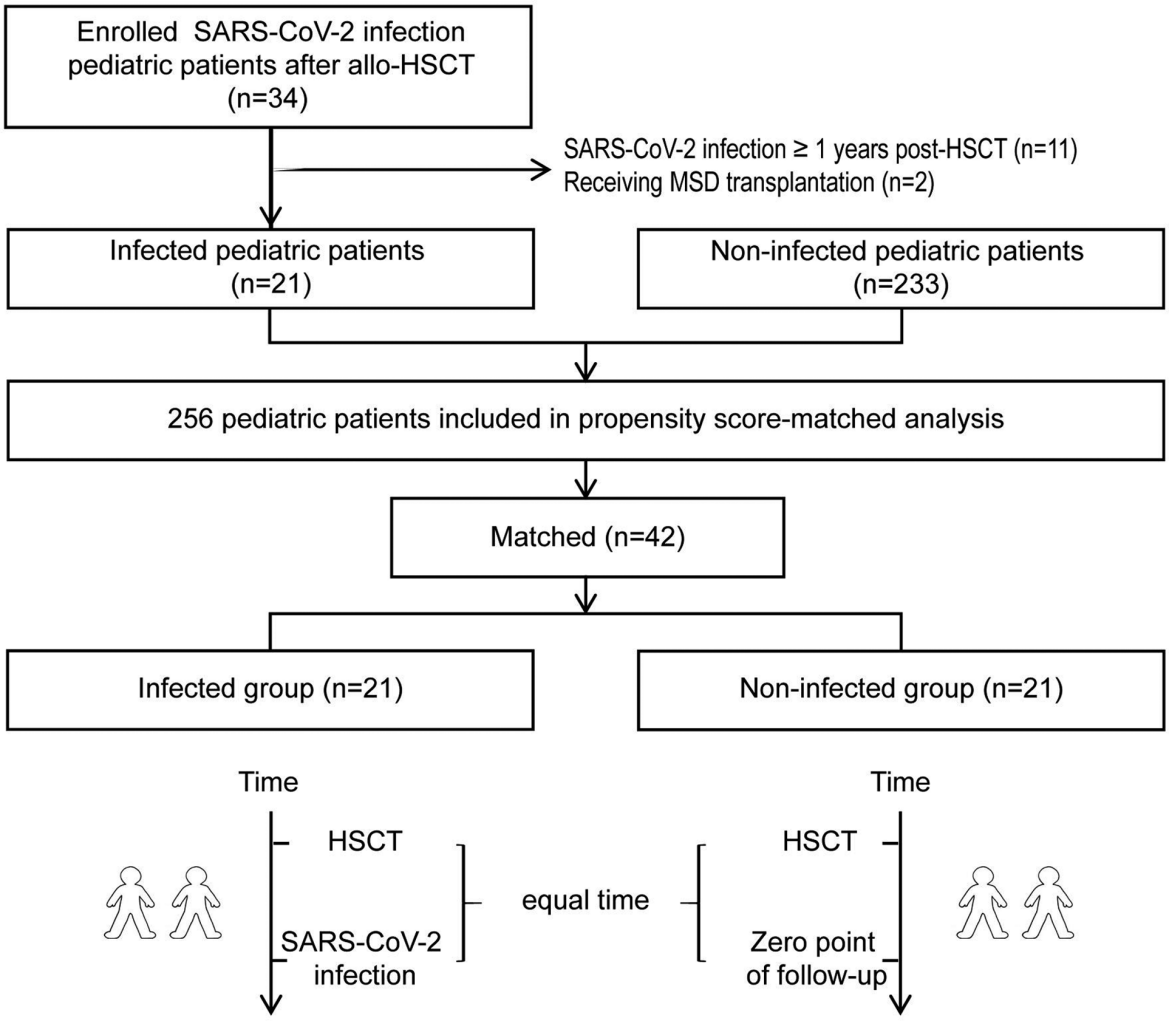
The flow chart of the retrospective study protocol.

As shown in Figure 1, we carried out to assemble infected patients with non-infected, historical children undergoing HSCT at our center. Matching was according to propensity score matching (1:1 basis, caliper 0.02) by five covariates: sex, age, pre-existing disorders, donor-recipient relationship and conditioning strategy. The time from allo-HSCT to SARS-CoV-2 infection in the infected group was used to determine the beginning of follow-up for the non-infected group, ensuring similar immune recovery status between the two groups. According to the daily 2019-nCoV nucleic acid testing conducted on all Chinese individuals, all of the enrolled patients were not infected with SARS-CoV-2 before HSCT, and neither were their donors.

### Primary end point

The primary endpoint of the study is to investigate the impact of SARS-CoV-2 on the IR kinetics of patients receiving different type of donors and age groups within 9 months after infection. Key immune parameters [count of CD3+ T cells, CD4+ T cells, CD8+ T cells, CD19+ B cells, and natural killer (NK, CD56+) cells] at baseline and month 1, 3, 6, and 9 were measured with flow cytometry. Monoclonal antibodies used were the classic T- (anti-CD3, CD4, CD8), B- (anti CD19 or CD20) and natural killer (NK)-cell antigens (anti-CD56 and CD16). Monoclonal antibodies were labeled by fluorescein-isothiocyanate (FITC), phycoerythrin, phycoerythrin-cyanin 5 or energy-coupled dye (ECD). Phenotypic analysis of lymphocyte subpopulation was performed on a Becton Dickinson FACSLyric flow cytometer; Data were calculated using cxp analysis software (Beckman-Coulter) on the basis of a FSC/SSC histogram or SSC/CD45 histogram. Lymphocyte subset reconstitution was defined as reaching the 10th percentile of age-matched reference values in two consecutive measurements (12).

### Secondary end point

The secondary endpoint of the study is the incidence of secondary infections and overall survival (OS) after SARS-CoV-2 infection. Secondary infections included viral (including cytomegalovirus, CMV and Epstein-Barr virus, EBV), bacterial, and fungal infections, whose diagnosis was based on clinical manifestation and laboratory examination. Real-time PCR was used to quantify DNA levels of CMV and EBV in plasma (13). Our center defined that the clinical cut-offs for a positive PCR result of CMV and EBV DNA were both set at 1,000 copies/ml. Bacterial and fungal infections were diagnosed by a new positive blood, sputum, and/or urine culture after SARS-CoV-2 infection.

### Statistical analyses

Data were expressed as the means and standard deviations (SD) or the medians and ranges, depending on the parametric or nonparametric distribution of the variable. The Fisher exact test was used to assess the categorical variables as appropriate, and the Mann-Whitney *U* test was used to assess the continuous variables. A two-tailed *P* value of >0.05 was considered statistically insignificant. The count of lymphocyte subsets in the infected group and non-infected group at different time points were compared using a repeated-measurement analysis of variance (RM-ANOVA). A multivariate Cox proportional hazards model was used to evaluate SARS-CoV-2 risk with each lymphocyte subset reconstitution. The cumulative incidence curve and Kaplan-Meier curve were used to assess the incidence of secondary infections and survival respectively. Statistical analyses were performed using R version 4.3.0.

### Ethical considerations

The study design and methods complied with the Declaration of Helsinki and were approved by the Ethics Committee and Institutional Review Board of the Institute of Hematology and Blood Diseases Hospital, Chinese Academy of Medical Sciences & Peking Union Medical College.

## Results

### Study population

We enrolled 42 consecutive patients, consisting of 21 in the infected group and 21 in the non-infected group, and the follow-up duration was 9 months. Baseline characteristics are presented in Table 1. The two groups had well balanced baseline characteristics. Despite the significantly higher proportion of children in the non-infected group who had chronic GVHD (cGVHD) at the time of infection compared to the infected group, the incidence of GVHD remained comparable between the two groups. The preexisting disorders included acute myeloid leukemia (AML), acute lymphoblastic leukemia (ALL), myelodysplastic syndromes (MDS), hybrid acute leukemia (HAL), and bone marrow failure (BMF). In the infected group, the average time from allo-HSCT to SARS-CoV-2 infection was 142 (17–347) days. 4.8% (*n* = 1/21) children were asymptomatic, 81.0% (*n* = 17/21) children were mild, 9.5% (*n* = 2/21) children were moderated and 4.8% (*n* = 1/21) children were severe. During SARS-CoV-2 infection, cough and fever were the most frequent symptoms, accounting for 61.9% (13/21) and 81.0% (17/21) respectively.

**Table 1:** Baseline characteristics of infected group and non-infected group post-propensity score matching.

	Infected Group	Non-infected Group	*P*
*n*	21	21	
Sex			1
Female	14 (66.7)	14 (66.7)	
Male	7 (33.3)	7 (33.3)	
Age, years			0.911
0–4	3 (14.3)	4 (19.0)	
4–12	12 (57.1)	11 (52.4)	
12–18	6 (28.6)	6 (28.6)	
Diagnose			1
Benign disease	4 (19.0)	4 (19.0)	
Malignant disease	17 (81.0)	17 (81.0)	
Conditioning			0.239
MAC	15 (71.4)	19 (90.5)	
RIC	6 (28.6)	2 (9.5)	
GVHD prophylaxis			1
CSA based	19 (90.5)	20 (95.2)	
TAC based	2 (9.5)	1 (4.8)	
Donor-recipient relationship			1
UCB	9 (42.9)	9 (42.9)	
HID	12 (57.1)	12 (57.1)	
Donor-recipient sex match grafts			0.119
Matched	12 (57.1)	6 (28.6)	
Dismatched	9 (42.9)	15 (71.4)	
ABO match graft			0.215
Matched	9 (42.9)	14 (66.7)	
Dismatched	12 (57.1)	7 (33.3)	
Cell compositions in graft, mean (SD)			
Infused nuclear cells,10^8^/kg	4.89 (4.42)	6.09 (5.54)	0.696
Infused CD34 + cells,10^6^/kg	2.63 (2.86)	1.43 (1.30)	0.379
Immunosuppressive therapy			1
Yes	19 (90.5)	20 (95.2)	
GVHD			
Yes	7 (33.3)	14 (66.7)	0.064
Acute GVHD			0.894
Grade, 1	3 (14.3)	4 (19.0)	
Grade, 2	1 (4.8)	1 (4.8)	
Grade, 3–4	1 (4.8)	2 (9.5)	
Chronic GVHD			0.003
Yes	2(9.5)	12(57.1)	

Values are reported as *n* (%) of patients unless indicated otherwise.

MAC, myeloablative conditioning; RIC, reduceintensity conditioning; CSA, cyclosporine A; TAC, tacrolimus; UCB, unrelated cord blood; HID, haploidentical donor; GVHD, graft vs. host disease.

### Immune reconstitution after SARS-CoV-2 infection

**Figure 2:**
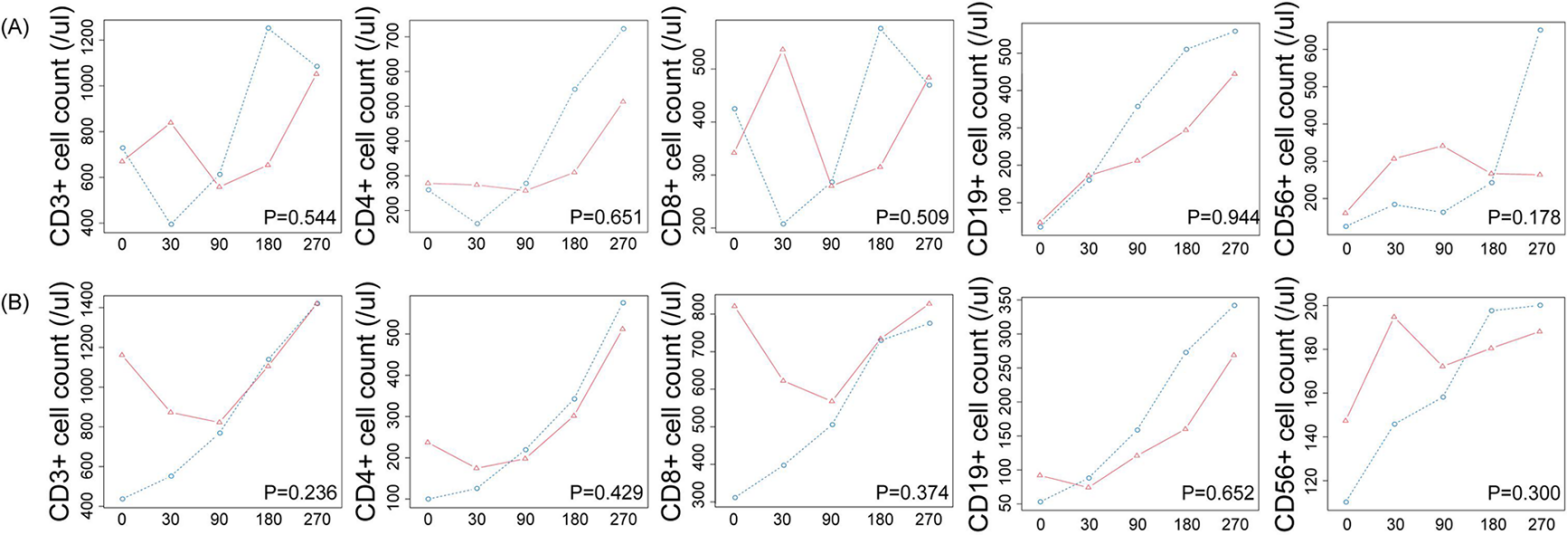
Time*group effects for the immune reconstitution of **(A)** UCB cohort and **(B)** HID cohort. 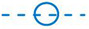: non-infected group; 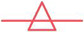: infected group.

In the UCB cohort, the NK cell reconstitution in the infected group was poorer compared to the non-infected group, but this difference did not reach statistical significance (*P* = 0.178). Overall, we observed similar recovery trends in UCB and HID recipients between the infected group and non-infected group during the 9 months follow-up. Figure 2 showed the lymphocyte reconstitution among the different donor cohorts.

Subsequently, we evaluated the associations of different lymphocyte subsets reconstitution with clinical factors using a Cox proportional hazards model. Clinical factors included SARS-CoV-2 infection, sex, age, diagnose, conditioning, donor, GVHD prophylaxis, ABO match graft, immunosuppressive therapy and GVHD. In univariate analysis, HID transplantation and ABO mismatched were identified as risk factors. Upon further multivariate analysis, while HID transplantation might suggest a trend towards poor CD19+ T-cell reconstitution [hazard ratio (HR): 0.43, 95% CI: 0.18–1.04, *p* = 0.06], there was no statistically significant difference.

### Clinical outcomes of SARS-CoV-2 infection

We evaluated the secondary infection, GVHD, and OS of the two groups. There were 6 viral (including 2 BK virus infection, 3 Cytomegalovirus infection and 2 EBV infection), 1 bacterial and 1 fungal infection occurred in the infected group after SARS-CoV-2 infection, while 4 viral (consisting with 3 BK virus infection and 2 Cytomegalovirus infection), 1 bacterial, and none fungal infections in the non-infected group. We observed no significant difference (*P* = 0.290) between 28.6% (*n* = 6/21) in the infected group vs. 14.3% (*n* = 3/21) in the non-infected group occurring secondary infection during follow-up. Besides, secondary infection is also similar between the infected and non-infected groups in either UCB cohort (*P* = 0.150) or HID cohort (*P* = 0.980).

**Figure 3:**
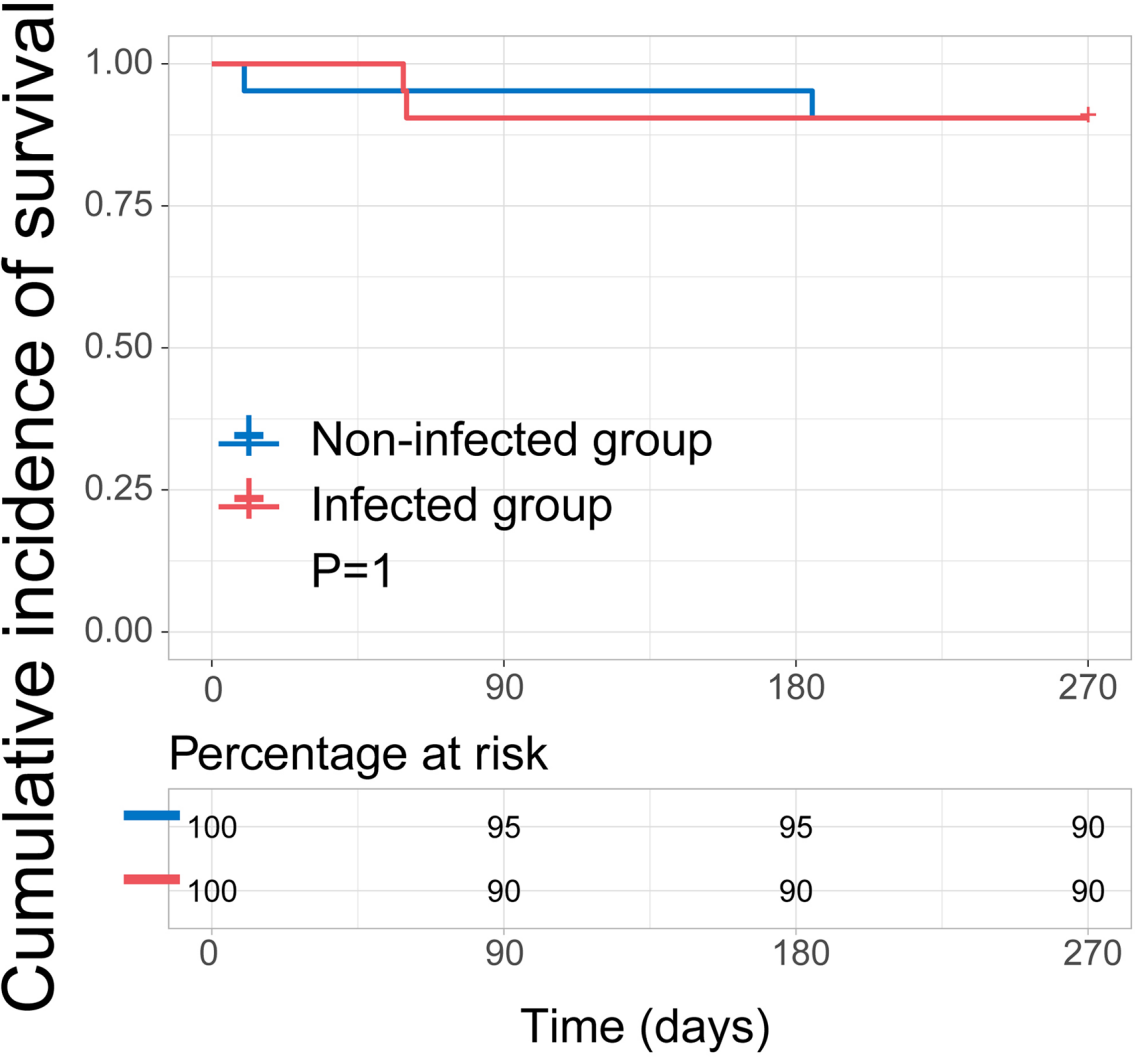
Kaplan-Meier curve of survival.

The 9-month OS was 90.5% (19/21) and 90.5% (19/21) in the infected group and non-infected group. In the infected group, all of tow children died from severe secondary infection and one of them developed disease relapse. The time from COVID-19 to death of them was 35 and 73 days. Moreover, two patient in the non-infected group died of a fatal secondary infection after relapse. OS did not differ significantly among the two groups (*P* = 1, Figure 3).

## Discussion

The degree of IR is critical to outcomes of post-HSCT patients. Nonetheless, the impact of SARS-CoV-2 infection on lymphocyte subset in post-HSCT children is scarce. We retrospectively analyzed the kinetics of IR and clinical outcomes of COVID-19 children in the authors' institution. Our primary focus was on the different lymphocyte reconstitution patterns of pediatric patients receiving different donors after COVID-19. Our analysis reveals that SARS-CoV-2 might temporarily impaired the IR process in the short term, with recovery to a comparable trend as observed in non-infected patients approximately 9 months post-infection.

T-cell and plasma cell deficiency is a typical profile of COVID-19, as well as other viruses such as EBV and CMV (7, 14, 15). Previous studies had shown that suggested that viremia caused by EBV, CMV and HHV6 impacts CD4+ T-cell reconstitution, while EBV and Human herpesvirus 6 (HHV6) viremia also influences CD8+ T-cell reconstitution (15, 16). In the case of SARS-CoV-2, it was able to affect hematopoietic compartment of the bone marrow and thymic atrophy, which correlated with the severity of infection (17). Complete and long-lasting T-cell reconstitution relies on *de novo* production of naive T cells in the thymus. The recovery of CD19+ B cells hange on the expansion of B lymphocyte progenitors within the graft, and their maturation necessitates the assistance of CD4+ T cells (18). In our study, the majority of enrolled children had mild COVID-19, aligning with previous research involving pediatric and early adolescent populations (12). Thus, we supposed that donor type might be a pivotal factor contributing to the varying patterns of IR. Given the low total lymphocyte count and HLA disparity in the graft (3), we speculated that the little impact of COVID-19 on UCB and HID cohorts may be difficult to detect due to their deficiency.

Interestingly, COVID-19 had no impact on the reconstitution of CD8+ T cells and NK cells in the analysis of various donor types. As key antiviral lymphocytes, the exhaustion of CD8+ T cells and NK cells is correlated with infection progression (19). Martin Schmidt-Hieber et.al found that low NK cell count (less than 161/microl) was significantly associated with CMV infection development (HR 2.92, *p* = 0.034) (20). Most children enrolled in our study had mild COVID-19, which may be one of the reasons for the observed result of these cells reconstitution. In addition, the antiviral immunity breaks down at an early stage post-infection and is quickly restored during the convalescent-phase (14, 19). A consistent finding from a retrospective analysis for patients post-autologous HSCT was a high level of CD8+ T cells after COVID-19 (21).

In conclusion, we found that SARS-CoV-2 likely affected IR in the short term for pediatric patients receiving UCB and HID recipients. Thus, we encourage the active application of anti-SARS-CoV-2 therapy and long-term monitoring for adolescents undergone transplantation. Future studies require a larger sample and longer follow-ups to determine the practical effect of SARS-CoV-2 on post-HSCT pediatric patients.

## Data Availability

The raw data supporting the conclusions of this article will be made available by the authors, without undue reservation.
